# Baduanjin is Better Balance Training Compared to Walking: A Cross-Sectional Study Based on Center of Gravity Trajectories

**DOI:** 10.7759/cureus.54620

**Published:** 2024-02-21

**Authors:** Weilan Jiang, Bingchen An, Shuangtao Liu, Chuan Xue, Chunlai Niu, Jie Qiu, Qilian Hu, Yaping Wang, Liang Chen, Liao Wang

**Affiliations:** 1 Department of Rehabilitation Medicine, Huadong Hospital Affiliated to Fudan University, Shanghai, CHN; 2 Department of Orthopedics, Shanghai Jiao Tong University School of Medicine Affiliated Ninth People's Hospital, Shanghai, CHN

**Keywords:** motion analysis, center of gravity, balance, walking, baduanjin

## Abstract

Background: It has already been demonstrated by previous studies that Baduanjin training can improve the body’s balance. However, its biomechanical mechanism remains unknown. Center of gravity (COG) trajectory analysis is an essential biomechanical test to explore the balance ability of the human body. Previous studies have not used the COG trajectory analysis technique to research Baduanjin training. The study utilizes COG trajectory analysis to analyze the trajectory of COG during Baduanjin training and compare it with that of walking, which is a common exercise for improving balance and aerobic ability, to determine if Baduanjin exercises affect the COG more than walking.

Materials and methods: Eight healthy female college students performed the walking and the eight forms of Baduanjin, a total of nine motions. The lower body kinematics were captured by the Vicon Motion Capture and Analysis System, while the kinetic data were acquired by the Kistler 3D Force Platform. The data were imported into Visual 3D to process the trajectory of the COG displacement amplitude, velocity, and acceleration of each motion. The COG horizontal envelope areas were calculated by Origin 9.0 Software (Origin Lab, Northampton, Massachusetts, USA) .

Results: Specific motions of Baduanjin provided significantly higher COG displacement amplitude, velocities, and acceleration training than walking. The F2 and F5 motions could provide a larger COG horizontal envelope area than walking. On the x-axis, F2 provided a greater COG displacement amplitude than walking, F1, F2, and F5 provided greater velocities, while all the motions provided greater accelerations. On the y-axis, all the motions except F2 provided greater COG displacement velocities and accelerations than walking. On the z-axis, F1-7 provided a greater COG displacement amplitude than walking, all the motions provided greater velocities, while all the motions except F2 provided greater accelerations.

Conclusion: Baduanjin training provides a more intense COG perturbation than walking, which may be a more challenging balance training than walking.

## Introduction

Baduanjin is a form of mind-body exercise, and its first recorded usage was during the Song dynasty (approximately 800 years ago) in China [1]. Baduanjin only has eight simple forms, which are linked together with smooth transitions from one form to the next [2]. A low demand for physical ability and training space makes it a home-based training for promoting physical health, even for frail people who are at risk of falls [3]. Baduanjin is also widely used in patient rehabilitation for stroke, Parkinson’s disease, knee osteoarthritis, diseases of the patellar extremity, etc [4]. Most of these studies have found that Baduanjin has the effect of improving the body’s balance and preventing falls [5-11].

Baduanjin is effective in improving balance function, even for elderly patients [6-8] as well as young people [9,10]. Su [9] observed female college students after eight weeks of Baduanjin training and found that their balance test scores improved significantly compared to pre-exercise. Feng [10] demonstrated that Baduanjin was more effective in improving the static balance ability of visually impaired young people compared with ordinary exercise training. Li [12] conducted an intervention to improve knee stability with Baduanjin and walking, revealing that Baduanjin had a positive effect on balance and gait indicators and significantly outperformed walking. Although Baduanjin exercises can effectively enhance balance better than walking, the mechanism remains unknown.

Center of gravity (COG) trajectory analysis is an essential biomechanical test to explore the balance ability of the human body. Xu and Song [13] analyzed the projection points of a human subject’s COG and found that the standard human COG displacement trajectory was generally spherically centered. If the area of the COG displacement trajectory was less than the normal range or the shaded areas were significantly unequal, then the balance function was considered impaired.

COG trajectory analysis is not only used for testing; adding COG perturbations is also an important means of balancing training. Urabe et al. [14] used virtual reality (VR) technology to induce swaying of the COG by allowing subjects to experience body tilting and loss of balance in a virtual space, indicating a significant increase in the subject’s ability to withstand external perturbations. Runge [15] found that increasing the platform velocity and greater COG interference during balance training might aid recovery of the balance function to a greater extent.

Previous studies have not used the COG trajectory analysis technique to research Baduanjin training. In our study, the COG trajectory was analyzed and compared during Baduanjin training and walking, which are common exercises for improving balance and aerobic ability, to illustrate that Baduanjin could provide greater balance training than walking [16]. The hypotheses are as follows: 1. Assess whether Baduanjin training results in greater COG displacement, velocity, and acceleration across the anterior-posterior, medial-lateral, and vertical planes as compared to walking. 2. Determine if practicing Baduanjin leads to a larger COG trajectory in horizontally shaded areas (an indicator of balance capacity) in comparison to walking. This research will provide insight into the potential biomechanical advantages of Baduanjin exercises for balance training, contributing to evidence-based recommendations for exercise regimens aimed at improving stability and reducing fall risk.

## Materials and methods

Subjects

Eight healthy female college student volunteers were recruited. The study inclusion criteria were as follows: (1) age was classified as 18-25, (2) body mass index (BMI) <30 kg/m^2^, (3) no history of lower limb fractures or trauma, no abnormalities, including knee abnormalities, hip abnormalities, lumbar spine deformities, etc. 

Testing procedure

**Figure 1:**
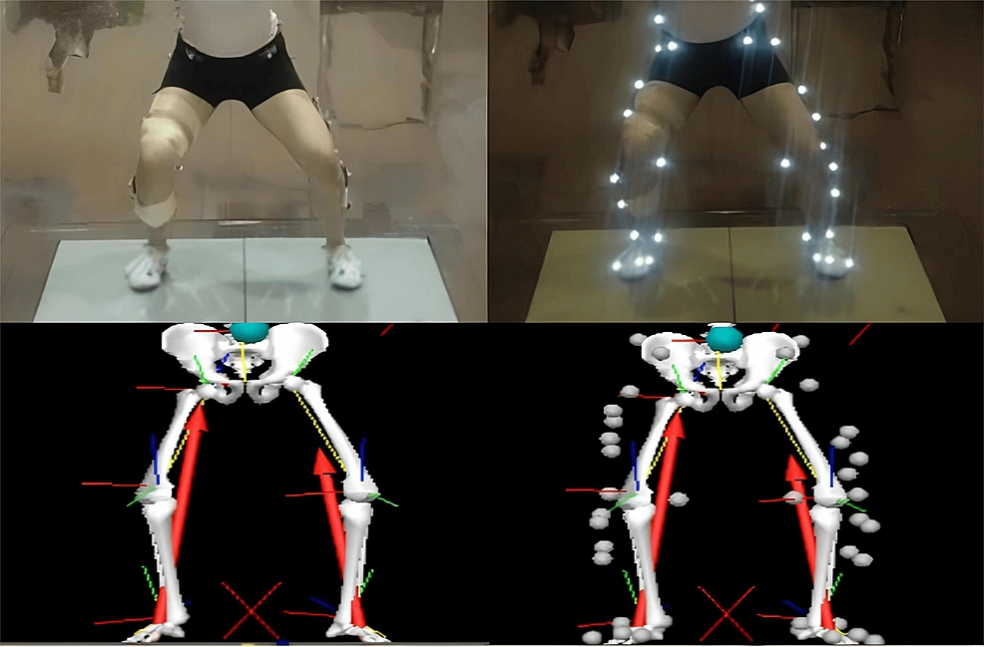
Reflective marker spheres (n=26) were placed at the corresponding bone landmarks according to the PCT protocol. PCT: point cluster technique.

Kinematic data were collected by 12 Vicon 3D infrared motion capture and analysis systems (200 Hz, Vicon Motion Analysis Inc., Oxford, UK). Twenty-seven reflective marker spheres were placed at the bony landmarks, including the highest point of the superior iliac spine, anterior superior iliac spine, posterior superior iliac crest, greater trochanter, medial and lateral femoral condyles, inner and outer ankle, heel, first and fifth metatarsals, and mid-thigh and mid-calf according to the ‘point cluster technique (PCT)’ protocol (Figure 1). Kinetic data were collected by two 3D force platforms (1000 Hz, Kistler Instruments AG Corp., Switzerland).

All subjects completed the testing of walking and the eight forms of standard Baduanjin exercise, according to the General Administration of Sport. Before the testing, the participants were guided by a professional instructor to learn the standard Baduanjin motions issued by the Fitness Qigong Management Center of the General Administration of Sports in China: Form 1 (F1). Prop up the sky with two hands to regulate tri-jiao, Form 2 (F2). Draw a bow on both sides like shooting a vulture, Form 3 (F3). Raise a single arm to regulate the spleen (Pi) and stomach (Wei), Form 4 (F4). Look back to treat five strains and seven impairments, Form 5 (F5). Sway the Head and Shake the Tail, Form 6 (F6). Pull toes with both hands to reinforce the kidney (Shen) and waist, Form 7 (F7). Clench one’s fist glare to and increase strength, Form 8 (F8). Rise and fall on tiptoe seven times to treat all diseases [2,17]. In the testing, the participants were instructed to walk at the fastest speed, to practice Baduanjin according to standard audio command speed and to step on one of the 3D force platforms with each foot for validation data. Then the gait and F1-8 motions were executed by lotting sequences. A two-minute break was taken between each motion test to avoid the influence of fatigue.

Data processing

The COG is one of the basic parameters reflecting the human body's morphological structure and mass distribution, which can provide a reference for medical rehabilitation, biomechanics, and other fields. In general, the COG is located 7 cm anterior to the superior border of the third sacral vertebra on the median side of the body in the standing position [18]. Zhou and Liu [19] and Lin [20] used a software package to obtain the COG position, curve, and acceleration to measure the balance ability of the walking process in healthy subjects and patients with hip arthritis.

The data were pretreated in Vicon Nexus software in C3D format and then imported into Visual 3D software (C-Motion, Inc., USA). First, a musculoskeletal rigid body model was established in Visual 3D containing 15 links for the half body. Secondly, the data were low-pass filtered (fourth-order, zero-lag Butterworth filter, cut-off frequencies of 10 Hz and 100 Hz). Finally, the COG data were obtained for each body part of the subject using the formula for calculating the COG:

$(x_{limb}=\tfrac{\sum_{s=1}^{L}P_{s}x_{cg_{s}}}{\sum_{s=1}^{L}P_{s}},y_{limb}=\tfrac{\sum_{s=1}^{L}P_{s}y_{cg_{s}}}{\sum_{s=1}^{L}P_{s}})$,

where L is the number of segments in the limb, xlimb and ylimb represent the limb’s center of gravity, xcg and ycg represent each segment’s center of gravity, and PS represents each segment’s mass proportion. The total body’s COG was computed similarly and was a weighted average of all body segments. That is:

$(x_{total}=\sum_{s=1}^{L}P_{s}x_{cg_{s}},y_{total}=\sum_{s=1}^{L}P_{s}y_{cg_{s}})$,

where xtotal and ytotal are the total body’s COG coordinates, xcg and ycg are the segments’ centers of gravity coordinates, and S is the number of body segments [21].

The 3D COG trajectory data were interpolated to 101 data points using linear interpolation in Origin 9.0 software (Origin Lab, Northampton, Massachusetts, USA) from the original data and solved for time with first-order differentiation to obtain the velocity variation and second-order differentiation to obtain the acceleration variation.

The walking cycle was defined as 0% when the right heel first hit the ground and 100% when the right heel hit the ground again. The Baduanjin movement cycle was defined as 0% at the moment of standing preparation and 100% at the moment of returning to an upright position, with all data taken from the subject’s dominant side. The walking's y-axis (anterior-posterior) COG displacement amplitude was calculated with the measuring point minus the average displacement points, and the y-axis COG velocity was calculated with a measuring point minus the average COG velocity. The trajectory of the subject’s COG was chosen on the x-axis (medial and lateral) and y-axis and plotted as a scatter plot, followed by using the 2D Confidence Ellipse plugin in Origin software to obtain the horizontal envelope area using Origin’s integral calculation function. The COG trajectory horizontal envelope area represents the shaded area of the COG trajectory enclosure, which provides a direct indication of the body's balance capacity [22].

Statistical analysis

The data were expressed as ‘mean ± standard deviation (mean ± SD)’, and the data were tested for normality and variance before comparison. Normally distributed data with a homogeneous variance were analyzed with one-way ANOVA. Data not in line with the normal distribution and homogeneous variance were analyzed with a non-parametric Kruskal-Wallis test, and post-hoc comparisons between groups were made with the Tukey method. All data statistical analyses were performed in Origin 9.0 software. Differences were considered significant when P < 0.05.

## Results

Subject characteristics

The subject characteristics are shown in Table 1.

**Table 1:** The subject characteristics. BMI: body mass index, BMI=weight/height^2^ (kg/m^2^).

Index	Distribution range
Number	8
Age	19.75 ± 1.98
Height (m)	1.60 ± 0.05
Weight (kg)	57.90 ± 7.72
BMI (kg/m^2^)	22.45 ± 2.09
Dominant side	Right

Comparison of the COG displacement amplitude and envelope area in Baduanjin training and walking

**Figure 2:**
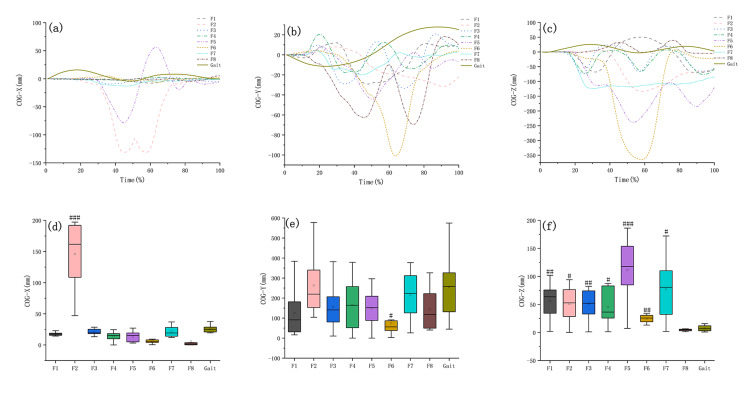
Comparison of the amplitude of COG displacement between Baduanjin and walking. Fn: the Nth movement of Baduanjin, x-axis: medial and lateral direction, y-axis: anterior and posterior direction, z-axis: vertical direction. (a)–(c) The trajectory of COG displacement in Baduanjin and walking. (d)–(f) The statistical differences between Baduanjin and walking in terms of maximum amplitude of COG displacement were #: p < 0.05, ##: p < 0.01, ###: p < 0.001. COG: center of gravity.

On the x-axis, F2 had the largest COG displacement amplitude (Figure 2a); on the y-axis, F6 had the smallest COG displacement amplitude (Figure 2b), while on the z-axis, F5 and F6 had the larger COG displacement amplitude (Figure 2c). The peak of COG displacement amplitude and statistical differences between the Baduanjin motions and gait are shown in Table 2 and Figures 2d-2f.

**Figure 3:**
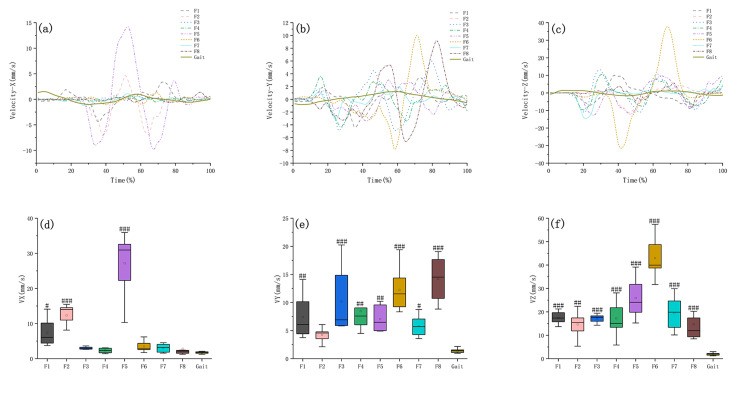
Comparison of the amplitude of the change and peak velocity of COG displacement in Baduanjin and walking. Fn: the Nth movement of Baduanjin, x-axis: medial and lateral direction, y-axis: anterior and posterior direction, z-axis: vertical direction. (a)–(c) The velocity curves of Baduanjin and walking. (d)–(f) The statistical differences between Baduanjin and walking in terms of the peak velocity of COG displacement were #: p < 0.05, ##: p < 0.01, and ###: p < 0.001. COG: center of gravity.

The body’s COG trajectory horizontal envelope is the area covered by the body’s COG sway trajectory on a horizontal plane [23]. As shown in Figure 3, F2 and F5 had envelope areas of 14255.24 mm^2^ and 20611.47 mm^2^, larger than the walking envelope area of 2352.52 mm^2^, while all other motions had a smaller envelope area than walking (Table 2).

**Table 2:** The peak of COG displacement amplitude, the COG trajectory horizontal envelope area and statistical differences between the Baduanjin motions and gait. Fn: the Nth movement of Baduanjin. The bold number indicates the highest peak value and the smallest P-value. COG: center of gravity.

Motion			F1	F2	F3	F4	F5	F6	F7	F8	Gait
COG displacement amplitude (mm)	x-axis	Peak value	17.57 ± 2.80	146.09 ± 53.88	20.92 ± 5.15	13.86 ± 7.60	13.58 ± 8.52	5.60 ± 3.03	21.43 ± 9.18	5.73 ± 11.94	25.84 ± 6.08
		P_Fn, Gait _	0.994	<0.001	1.000	0.943	0.936	0.482	1.000	0.491	/
	y-axis	Peak value	125.9 ± 127.12	263.34 ± 159.79	156.03 ± 115.68	165.74 ± 129.56	149.46 ± 98.50	74.74 ± 69.86	215.86 ± 120.93	143.31 ± 110.79	256.45 ± 164.23
		P_Fn, Gait_	0.487	1.000	0.781	0.856	0.722	0.020	0.998	0.662	/
	z-axis	Peak value	56.45 ± 33.01	51.23 ± 31.96	50.26 ± 28.32	45.70 ± 31.42	97.30 ± 65.29	24.74 ± 7.42	77.55 ± 55.74	4.51 ± 1.86	7.53 ± 5.65
		P_Fn, Gait_	0.009	0.012	0.009	0.015	<0.001	0.002	0.003	< 0.005	/
COG trajectory horizontal envelope area (mm^2^)		Value	1816.64 ± 1256.88	14255.24 ± 10245.78	2479.62 ± 1915.92	975.59 ± 495.30	20611.47 ± 14684.76	3084.18 ± 667.70	1230.62 ± 921.93	1462.78 ± 521.89	2352.52 ± 1388.96
		P_Fn, Gait_	0.463	0.010	0.889	0.042	0.007	0.233	0.064	0.139	/

Comparison of the COG displacement velocity and acceleration in Baduanjin training and walking

**Figure 4:**
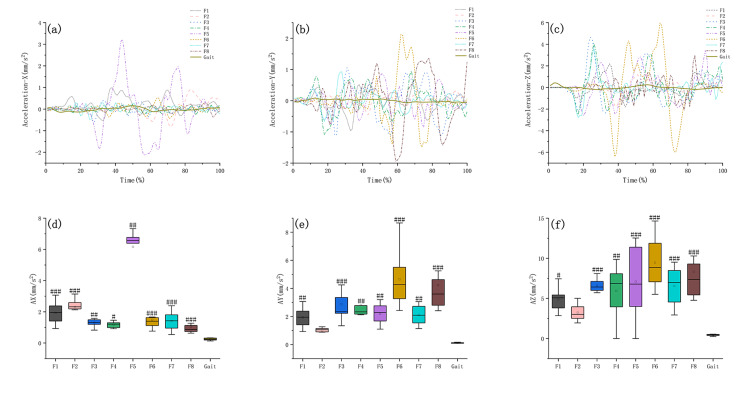
Comparison of the amplitude of change and peak acceleration of the COG displacement in Baduanjin and walking. Fn: the Nth movement of Baduanjin, x-axis: medial and lateral direction, y-axis: anterior and posterior direction, z-axis: vertical direction. (a)–(c) The acceleration curves of Baduanjin and walking. (d)–(f) The statistical differences between Baduanjin and walking in terms of peak acceleration of COG displacement were #: p < 0.05, ##: p < 0.01, ###: p < 0.001. COG: center of gravity.

On the x-axis, F5 had the greatest COG displacement velocity and acceleration (Figures 3a, 4a); on the y-axis, F6 had the greatest COG displacement velocity and acceleration (Figures 3b, 4b), while on the z-axis, F6 had the greatest COG displacement velocity and acceleration (Figures 3c, 4c). The COG displacement velocity and acceleration and statistical differences between the Baduanjin motions and gait are shown in Table 3, Figures 3d-3f, and Figures 4d-4f.

**Table 3:** The peak of COG displacement amplitude, acceleration and statistical differences between the Baduanjin motions and gait. Fn: the Nth movement of Baduanjin. The bold number indicates the highest peak value and the smallest P-value. COG: center of gravity.

Motion			F1	F2	F3	F4	F5	F6	F7	F8	Gait
COG displacement velocity (mm/s)	x-axis	Peak value	7.39 ± 3.70	12.32 ± 4.01	2.89 ± 0.54	2.37 ± 0.65	27.16 ± 9.44	3.44 ± 1.45	3.04 ± 1.18	2.79 ± 2.55	1.82 ± 0.47
		P_Fn, Gait_	0.041	<0.001	0.996	1.000	0.002	0.967	0.992	0.462	/
	y-axis	Peak value	7.39 ± 3.70	4.25 ± 1.19	10.17 ± 5.94	8.46 ± 4.52	7.03 ± 2.20	14.21 ± 3.88	5.80 ± 1.84	12.25 ± 3.75	1.41 ± 0.39
		P_Fn, Gait_	0.001	0.392	<0.001	0.001	0.005	<0.001	0.038	<0.001	/
	z-axis	Peak value	17.42 ± 2.50	14.64 ± 5.27	17.19 ± 1.67	17.22 ± 7.18	25.88 ± 7.92	42.99 ± 8.09	19.48 ± 7.14	14.71 ± 7.92	1.96 ± 0.58
		P_Fn, Gait_	<0.001	0.001	<0.001	<0.001	<0.001	<0.001	<0.001	<0.001	/
COG displacement acceleration (mm/s^2^)	x-axis	Peak value	1.93 ± 0.70	2.31 ± 0.56	1.30 ± 0.25	1.18 ± 0.19	6.17 ± 1.41	1.49 ± 0.64	1.41 ± 0.62	1.05 ± 0.53	0.25 ± 0.07
		P_Fn, Gait_	<0.001	<0.001	0.002	0.011	0.002	<0.001	<0.001	<0.001	/
	y-axis	Peak value	1.93 ± 0.70	1.00 ± 0.25	2.87 ± 1.43	2.82 ± 1.18	2.18 ± 0.73	4.65 ± 2.05	2.12 ± 0.69	4.23 ± 2.28	0.12 ± 0.03
		P_Fn, Gait_	0.008	0.493	<0.001	0.001	0.003	<0.001	0.003	<0.001	/
	z-axis	Peak value	4.87 ± 1.40	3.24 ± 1.08	6.61 ± 0.82	2.82 ± 1.18	5.96 ± 3.21	9.47 ± 3.16	6.56 ± 2.39	8.34 ± 4.20	0.44 ± 0.10
		P_Fn, Gait_	0.017	0.340	<0.001	0.001	<0.001	<0.001	<0.001	<0.001	/

## Discussion

Balance training is a workout to improve motor function and avoid injury from falling [24]. Adding COG perturbations exercise in safety conditions is a common method of balance training. Previous studies have relied on slow or less challenging tasks for safe balance training [25]. However, it was thought that even for a frail elderly person, a more challenging balance exercise should be recommended under individual supervision to improve postural balance [25]. The greater the COG amplitude, velocity, and acceleration of the balance perturbation, the more benefit for the balance training, provided conditions are safe and tolerable for different conditioning [26]. In this study, we discovered that specific motions of Baduanjin provided significantly higher COG displacement amplitude, velocity, and acceleration training than walking, suggesting that Baduanjin can provide more challenging balance training than walking.

The envelope area of the COG displacement trajectory can reflect the degree of balance ability, i.e., the degree of sway [27]. Cho et al. [28] used virtual reality-based balance training to exercise the balance of elderly people, with the result that the area of the envelope covered by the center of pressure of the subjects was significantly reduced compared to the area before training, demonstrating an improvement in the balance ability. In this study, it could be seen that the F2 and F5 of Baduanjin provided a larger COG horizontal displacement envelope area during exercise than walking, allowing a subject to adapt to a greater range of sway in the trajectory of the COG, which proves to be more intense balance training than walking. On the horizontal plane, F2 can be used to train sidestepping strategies, which are particularly useful for improving balance, coordination, and lateral stability since F2 is a sidestep movement [29], while F5, with no movement of lower limbs relative to the ground, is more suitable for static balance training. 

Balance disorders may manifest as problems with balance function in different directions; e.g., Parkinson's manifests as balance disorders in the anterior-posterior direction and medial-lateral direction [30]. Therefore, targeted training based on balance disorders is required. Our result showed that in the medial-lateral direction (x-axis), F2 provided a significantly greater COG displacement amplitude than walking, F1, F2, and F5 provided significantly greater COG displacement velocities than walking, while all the motions provided significantly greater COG displacement accelerations than walking. As mentioned above, F2 and F5 had larger envelope areas than those of the walking. Consequently, for individuals with instability in the medial-lateral direction, we recommend focusing on F2 training since it can provide greater COG displacement amplitude, velocity, and acceleration in this direction, as well as a larger envelope area.

In the anterior-posterior direction (y-axis), the COG displacement amplitudes of F1, F2, F3, F4, F5, F7, and F8 were not statistically different from those of walking, while the peak COG displacement amplitude of F6 was smaller than that of walking, which had a statistical difference. All the motions except F2 provided significantly greater COG displacement velocities and accelerations than walking. Further analysis revealed that F6 had the largest peak COG velocity and acceleration in the anterior-posterior direction. Given that F5 simultaneously had a larger envelope area than that of the walking, we recommend focusing on training F5 in the anterior-posterior direction to improve balance deficits.

In the vertical direction (z-axis), F1-7 provided a significantly greater COG displacement amplitude than walking. All the motions provided significantly greater COG displacement velocities, while all the motions except F2 provided significantly greater COG displacement accelerations than walking. However, the COG displacement amplitude of F8 was smaller than that of walking, which had a statistical difference. Given the above results, all the motions except F2 and F8 can be used to improve balance deficits in the vertical direction.

We also acknowledge several limitations to our study. First, walking speed can confound biomechanical gait analysis, and we standardized speed by measuring the point minus the average COG velocity when processing COG velocity data for walking on the y-axis. Future studies can use more complex and precise methods like the per-sample interpolation method (IP method) and the PCA interpolation method (PCA method) to eliminate interference with walking speed when comparing biomechanical data at different walking speeds. Second, our study only included healthy female individuals as subjects, and the sample size was small. Future studies should include patients suffering from stroke, Parkinson’s, arthritis, etc., as subjects and incorporate a larger sample size. Finally, our study provides evidence that Baduanjin training is a more challenging balance exercise than walking, which requires more clinical validation in the future. Despite these limitations, our study suggests that Baduanjin exercise can be an effective alternative to walking for individuals looking to improve their balance.

## Conclusions

Certain movements of Baduanjin exercises had greater training on the COG trajectory in the anterior-posterior direction, medial-lateral direction, and vertical direction than normal walking, as evidenced by higher displacement, velocity, and acceleration. This indicated that the Baduanjin exercises provided more intense training for the subjects' COG disturbances. We recommend focusing on F2 training for individuals with balance instability in the medial-lateral direction, F5 in the anterior-posterior direction, and all the motions except F2 and F8 in the vertical direction. Based on these findings, tailoring Baduanjin training to each patient's specific needs may lead to better clinical outcomes.
